# Mapping three-dimensional temperature in microfluidic chip

**DOI:** 10.1038/srep03321

**Published:** 2013-11-25

**Authors:** Jinbo Wu, Tsz Yan Kwok, Xiaolin Li, Wenbin Cao, Yu Wang, Junying Huang, Yaying Hong, Dongen Zhang, Weijia Wen

**Affiliations:** 1 Department of Physics, the Hong Kong University of Science and Technology, Clear Water Bay, Kowloon, Hong Kong; 2 Nano Science and Nano Technology Program and Department of Physics, the Hong Kong University of Science and Technology, Clear Water Bay, Kowloon, Hong Kong

## Abstract

Three-dimensional (3D) temperature mapping method with high spatial resolution and acquisition rate is of vital importance in evaluating thermal processes in micro-environment. We have synthesized a new temperature-sensitive functional material (Rhodamine B functionalized Polydimethylsiloxane). By performing optical sectioning of this material, we established an advanced method for visualizing the micro-scale 3D thermal distribution inside microfluidic chip with down to 10 ms temporal resolution and 2 ~ 6°C temperature resolution depending the capture parameters. This method is successfully applied to monitor the local temperature variation throughout micro-droplet heat transfer process and further reveal exothermic nanoliter droplet reactions to be unique and milder than bench-top experiment.

Temperature as a fundamental and basic characterization for any thermal energy process demand researchers to strive their capability to push its probing technique to the limit, namely fast responsive real-time monitoring with high accuracy, excellent spatial and temperature resolution^1^,^2^. Heat transfer or heat loss to surrounding for macro object might be insignificant to the process of interest while for micro object (from μm to nm) due to high surface-area-to-volume (SAV) ratio, heat loss becomes an issue. Recent developments in droplet-based microfluidics designated droplet generation manipulation on small devices called Lab-on-a-Chip (LOC)^3^. It has great promise in enabling integrations of multiple steps of complex reactions without having the chip size and complexity increased^4^,^5^,^6^. In practical, the size of micro-droplet being manipulated in a LOC device ranging from nano- to picoliters in size, temperature variation inside such micro-droplet reflects the thermodynamics of the reaction. Therefore, droplet temperature measurement promotes better understanding of chemical reactions thermodynamics in micro-scale and the establishment of novel LOC platform.

For different process or system scale, temperature probing techniques vary to accommodate experiment requirements. In microfluidics, ideal temperature measurement technique should have following criteria: (1) high spatial and temperature resolution, (2) high acquisition rate, (3) inertness in solvent, as well as (4) photo- and heat- resistance. Unfortunately, such ideal probing method is rare in today technology. Traditional embedded thermocouple and thin-film resistor are common approaches and easily implemented in microfluidics^7^,^8^. However, they are confined to a single location and not capable of characterizing full-field temperature. Raman spectroscopy and fluorescence thermometry are both noncontact and nondestructive *in situ* detection techniques. Raman spectroscopy is well suited for measurement from room temperature (RT) to above 1000°C, its spatial resolution can be as small as 500 nm using a high-magnification object^9^. Yet it measures only one point at a time at slow capturing speed of 0.5 point per second and thus low acquisition rate^10^. Fluorescence thermometry became popular for both continuous flow and droplet microfluidics. It is an easily accessible and full-field technique, which is also capable of mapping temperature over a large area with high acquisition rate^10^,^11^,^12^,^13^. Rhodamine B (RhB), added and dissolved into droplets, performed droplet temperature measurement based on its temperature-dependent fluorescence intensity or fluorescence lifetime^11^,^14^. Although RhB offers excellent submicrometer spatial and microsecond temporal resolution, absorption of RhB in Polydimethylsiloxane (PDMS), a widely used polymer for LOC devices, or chemical solvent, thermal-bleaching and photo-bleaching are three major obstacles for RhB to obtain higher accuracy and precision and consequently restrains its application in measuring temperature^13^,^15^. Besides, addition of RhB to liquid/droplet for temperature measurement may alter experimental outcomes. Thus a new technology solving these problems is on demand.

In this study, by grafting RhB to PDMS with allyl glycidyl ether (AGE), we have created an ingenious temperature-sensitive functional material, RhB-AGE-PDMS (RAP), for temperature sensing in LOC devices. We applied RAP in a LOC device to perform three-dimensional (3D) temperature mapping using confocal microscope, where the fluorescence quantum efficiency of RAP was adopted as a temperature-dependent variable. Outstanding solvent, thermal stability and photo stability of RAP permits accurate and rapid temperature measurement of local temperature surrounding a nanoliter (nL) droplet trapped inside a RAP chamber. This new thermometric methodology is sufficient to characterize vigorous reaction in nL droplet which is much safer and later on recognized that behave distinctively from bench-top size experiments in mL volume.

## Results

**Figure 1:**
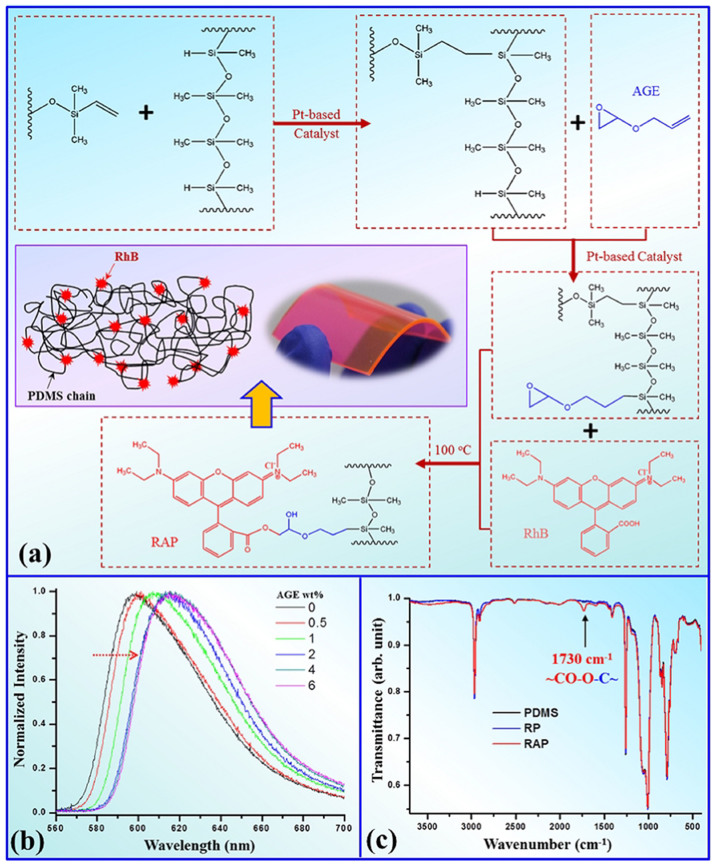
(a) Synthetic Route of RAP: (i) PDMS polymerization through platinum-catalyzed hydrosilylation reaction; (ii) The vinyl group of AGE reacted with the silyl (SiH) group of PDMS chain through platinum-catalyzed hydrosilylation reaction; (iii) The epoxy group of AGE-PDMS reacted with carbonyl group of RhB through ring open reaction. The inset is a schematic illustration of RAP molecular chain: PDMS main chain with RhB grafted on the side chain and a snapshot of the RAP. (b) Fluorescence emission of spectra of RAP with different AGE concentration in wt%. (c) FT-IR spectra of PDMS, RP and RAP obtained from optimized conditions.

RhB do not have any functional group that can react with PDMS, therefore AGE was chosen as a molecular connector between them (Figure 1a). AGE and RhB was added to PDMS mixture while PDMS is partly polymerized (reaction i)^16^. The remaining Si-H reacted to the vinyl group of AGE through platinum-catalyzed hydrosilation (reaction ii)^17^. After PDMS is fully cured at RT, RAP was heated to complete the final step. In this step, RhB was linked to PDMS through opening the epoxy ring (reaction iii)^18^,^19^.

The UV-Vis absorption and fluorescence emission spectra of RAP with 0.5 wt% of AGE at various incubation times are shown in Figure S1. The absorption peaks decrease from RAP 1 to 3 suggested that the reduction of the RhB concentration is caused by RhB aggregation after ethyl alcohol evaporated, while RhB diffused gradually in PDMS and decomposed during incubation at 100°C^20^. The absorption peaks become narrower from RAP 1 to 6 while keeping at the same absorbance from 5 to 6 indicated that the molecular structure of RhB became homogeneous and concentration of RhB did not decrease. These phenomena suggested that RhB was grafted onto PDMS gradually and become stable after attaching to PDMS. As it can be seen from the inset phase contrast and fluorescence images of RAP microfluidic chip, RhB was homogeneously dispersed and grafted onto PDMS matrix. The maximum emission wavelength of RAP 1 ~ 6 were blue shifted. This confirms RhB aggregation during mixing, and diffusion while incubating, since the emission peak of lower concentration was shifted to lower wavelength^21^. Nevertheless, the conjugated *π*-electron system of fluorescence in RhB molecule was not interrupted after grafting, generating PDMS-based polymer with fluorescent properties successfully. Figure 1b shows the fluorescence spectra of RAP with different AGE concentration under the same incubation time (25 hours) and RhB concentration (0.02 wt%) for comparison. The emission peaks of RAP were red shifted gradually from 598 to 615 nm after AGE concentration increased to 2%. This red shift further confirms that new chemical bonds in RhB were formed as shown by other authors^19^. In addition, when more than 4 wt% AGE is added, we observed the RAP cannot fully cure at RT even for several weeks. Therefore we concluded the optimized incubation time, RhB and AGE concentration are 25 hours, 0.02 wt% and 2 ~ 4 wt%, respectively, under which do not hamper the mechanical property and bonding force of RAP microfluidic chip and will be maintained throughout the experiment. One remarkable result should be noted that a new peak (1730 cm^−1^) appeared on the FT-IR spectrum of optimized RAP (Figure 1c) which corresponded to the stretching vibration of C = O of an ester group. This peak was not found in physical mixture of RhB-PDMS (RP). These FT-IR analysis, absorption and fluorescence spectra are all evidence which support the grafting of RhB onto PDMS was tremendously successful.

To test the chemical stability of the RAP, a slab of RAP was soaked in 5 common solvents in microfluidics: water, Silicone oil, acetone (polar), hexadecane (nonpolar) and ethyl alcohol. The experimental results are given in Figure S2a. It is noticeable that RAP fluorescent intensity changes slightly (less than 5%) after 2 hours of solvent soaking. Figure S2 (b–c) showed two plots of the normalized fluorescence intensities of RAP as a function of temperature and time (subjected to light or heat) at three different temperatures (24.5, 59.5 and 99.5°C). The fluorescence intensity decreased linearly with increase in temperature (from 20 to 100°C). The calibrated intensity-temperature relationship can be adapted to characterize temperature inside a RAP chip. In contrast, the fluorescence intensity remained fairly constant with continuous light and thermal exposures. A proposed reason for this photostability and thermal stability is that, transformation from cationic to lactone form and further decomposition are prohibited by grafting RhB onto PDMS^18^,^20^. The cationic molecular is strongly colored and emissive while the second lactone-containing molecular is colorless and exhibited no emission^19^.

Local heat and temperature variation was externally induced by black ink droplets inside a microfluidic chip, where the droplets were wirelessly and locally heated by an IR laser. A droplet generation device which has the ability to program the droplet number, volume and sequence, precisely and digitally through micro-sampling technique, was combined with a RAP chip. Detailed setup is described in Figure S3.

**Figure 2:**
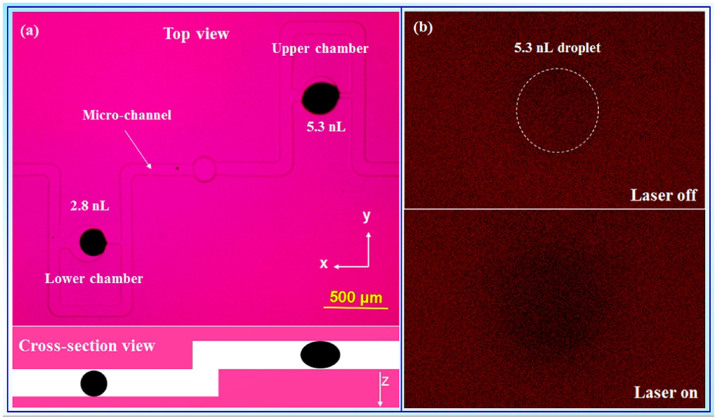
(a) Top and schematic cross-section views of microfluidic chip for droplet capture. (b) Fluorescence images taken from confocal microscope under the 5.3 nL droplet when laser is off and on.

The top and cross-section views of a RAP microfluidic chip for droplet capture are displayed in Figure 2a. The droplet-trapping compartment is designed on the basis of the hydrodynamic model^22^. Two ink droplets, which were generated to approximately 5.3 and 2.8 nL from the sampling device, were guided into the RAP chip. They are trapped in the upper and lower compartments respectively and then heated by the IR laser. Figure 2b displays fluorescence images (xy plane) of the RAP chip underneath the 5.3 nL droplet taken from the confocal microscope. When the IR laser was on, the black ink droplet absorbed the IR energy which converted into heat energy and caused the droplet temperature to rise. As expected, due to the local increase in temperature, RAP fluorescence intensity decreased in the vicinity of the droplet after the IR laser was turned on.

**Figure 3:**
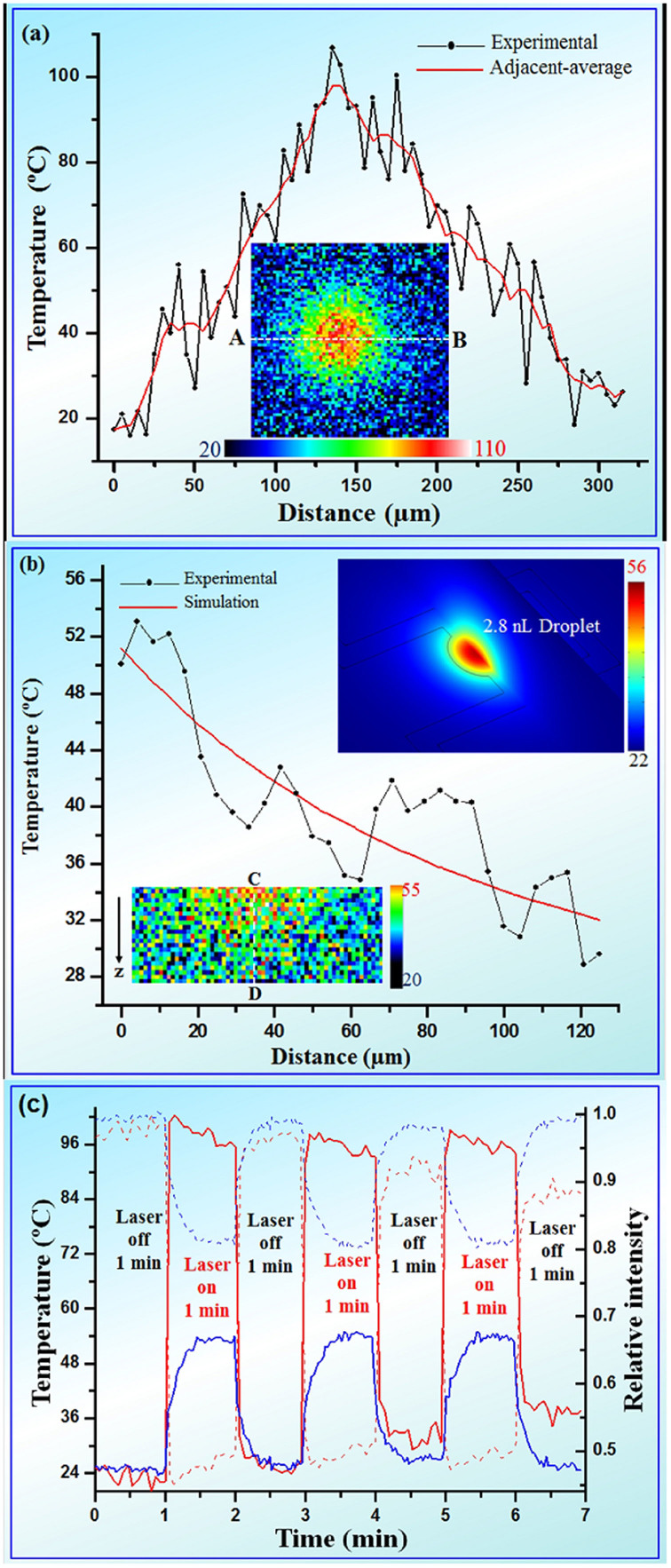
Temperature distributions along different dimensions. (a) Temperature distribution along AB line in the inset. Black line is experimental result. Red line is smoothed data by adjacent-average. The inset is temperature map (xy plane) under 5.8 nL droplet when laser is on. (b) Temperature along CD line as a function of distance to 2.8 nL black ink droplet. Black and red lines are experimental and simulation results respectively. The lower inset is thermal map (xz plane) under 2.8 nL droplet. The upper inset is a snapshot of 3D temperature distribution of 2.8 nL in simulation. (c) Variation of central temperature (solid line) and relative fluorescent intensity (dash line) along time for 2.8 nL (blue line) and 5.3 nL (red line) black ink droplet.

The brightness of each pixel in the image represents the intensity of RhB fluorescence at corresponding location in the RAP chip. Based on the calibrated temperature-intensity relationship, the relative changes in fluorescence intensity were converted to temperature values and were mapped in the inset of Figure 3a. The temperature profile along line AB was plotted in Figure 3a. Large fluctuation comes mainly from the instability of the photomultiplier tube (PMT) photo-detector and exciting laser during capturing in different times and the heterogeneous dispersion of RhB in PDMS. Assuming that the RhB is homogeneously dispersed in PDMS, the same location is excited with same laser intensity and the emission photo captured in PMT was converted to brightness in the same multiple along time, no fluctuation or noise would be observed. However, the RhB is not homogeneously dispersed in PDMS, and our confocal laser intensity and PMT was not stable along time in high scanning rate. The fluctuation could be diminished by reducing the scanning rate or increasing the laser intensity. It is also noticed that in Figure 3a, large fluctuation could be eliminated by adjacent-average. So we estimated that the spatial resolution along xy plane was 5 um after adjacent-average in Figure 3a. Temperature reached a central maximum in the line profile (Figure 3a), which is then defined as central temperature of the ink droplet. In the present confocal microscope, from the level of noise, the temperature and temporal resolution is estimated to be 2 ~ 6°C and 550 ms, while maintaining at the same temperature and temporal resolution, horizontal spatial (xy plane) resolution. On the other hand, one can always adjust capture parameters (such as pinhole size, pixel size) of the confocal microscope to maximize among temperature, temporal and spatial resolutions to accommodate different needs.

With the use of confocal microscope, RAP-based fluorescence thermometry can be used to perform axial temperature measurement. Fluorescence images underneath the 2.8 nL droplet were captured along z direction before and after heating the droplet to obtain the net fluorescence gain. We converted the net fluorescence gain of the central spot and then plot its axial temperature along z direction on Figure 3b. To verify the accuracy of measured temperature and gain further insight into 3D thermal distribution of the behavior of heat diffusion, a finite element model (FEM) of the RAP microfluidic chip was implemented using COMSOL software. Figure 3b shows the experimental result and simulation of temperature profile along the line CD, which is the vertical line profile of the central maximum. The upper inset in Figure 3b is a snapshot of 3D temperature distribution of 2.8 nL in simulation. Detailed information is provided in Supplementary Information. The experimental temperature (central temperature) distributions exhibit the same decreasing trend with simulation value in z direction despite significant noise is presented. This suffers from low signal intensity and high gain in the camera system to capture the fluorescence, which caused by relative small pinhole used for the narrow section thickness (10 μm) and thick chip substrate (~2 mm). The temperature and spatial resolution in axial direction is limited to be 3 ~ 8°C and 10 μm, respectively, in the present state but could be significantly improved by higher RhB concentration, higher-NA objectives, and thinner chip substrate.

Furthermore, the droplet temperature could be estimated from the highest central temperature (closest to the droplet). However, the highest central temperature is supposed to be lower than the droplet temperature since the droplet is surrounded by the carrier fluid (Si oil in our case)^23^ which is indirectly contacting with channel surface. It is instructive to know the divergence between our result inferred by RAP and the true droplet temperature. FEM simulations were therefore performed to calculate the temperature divergence numerically. The result of the central temperature as a function of droplet temperature is plotted in Figure S4. Figure 3c shows variation of central temperature (solid line) and relative fluorescent intensity (dash line) closest to 2.8 nL (blue line) and 5.3 nL (red line) black ink droplet. The real-time central temperature reflects the local heat energy dissipation of the droplet which is determined by heat energy, interfacial properties, specific heat and mass of the droplet. Hence, we can monitor the central temperature to investigate the local heat production and dissipation of the micro-droplet in such a micro-environment.

To investigate the relation between heat generated and transfer rate, a preliminary calculation is derived. Assuming the IR laser used has a uniform light spot and a stable power output, the rate of heat converted from IR by the micro-droplet is: 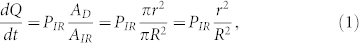
where P_IR_ is the IR power, A_D_ and A_IR_ are the area of the droplet and laser point, r and R are the radius of the droplet and IR laser point. The heat generated by the droplet will dissipate to surrounding by conduction and carrier flow advection. Considering Fourier's law for advective and conductive heat dissipation respectively and assuming that the droplet temperature is uniform, we have the following equation: 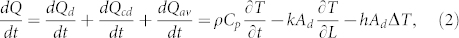
where Q_d_ is the heat energy holding in droplet, Q_cd_ and Q_av_ are the heat energy conducted and advected to the surrounding carried fluid, ρ is the mass density, C_p_ is the specific heat capacity and T is the temperature of the droplet, t is the time variable, k is the thermal conductivity of carried fluid, A_d_ the heat transfer surface area (droplet surface area), L the distance from droplet to the surrounding carried, h is heat transfer coefficient and ΔT is the temperature difference between the droplet and the surrounding carried fluid. The radius of IR laser point, 2.8 and 5.3 nL droplets are 1, 0.095 and 0.13 mm, so the 2.8 and 5.3 nL droplets received 27 and 51 mJ of the heat energy per second, respectively. After the IR laser was turned on, the heat energy in the droplet is constantly produced from IR absorption and continuously lost to the environment. At the beginning, the heat lost rate is smaller than heat production due to small temperature difference between the droplet and environment, so that dQ_d_/dt > 0 and the droplet temperature increased. For the 2.8 nL droplet, the central temperature climbed gradually up. Finally, the heat loss rate increased and finally equaled to heat production rate, and namely they are in dynamic equilibrium. Thereby the droplet temperature reached to a plateau (~53°C), as observed in the Figure 3c. Interestingly, the central temperature of the 5.3 nL black ink droplet rapidly reached a peak (~100°C) and then began to drop. It is believed that the droplet evaporated at elevated temperature and shrunk, therefore the IR absorption decreased. As the droplet temperature increased, the heat loss increased. After IR laser was turned off, the temperature dropped quickly (in ~8 seconds) to around 36°C and then cool down gradually to RT. The active and localized IR heating and passive cooling rate are up to 20 and 10°C/second respectively, which could be used to animate rapid temperature cycling for amplification of DNA molecules.

**Figure 4:**
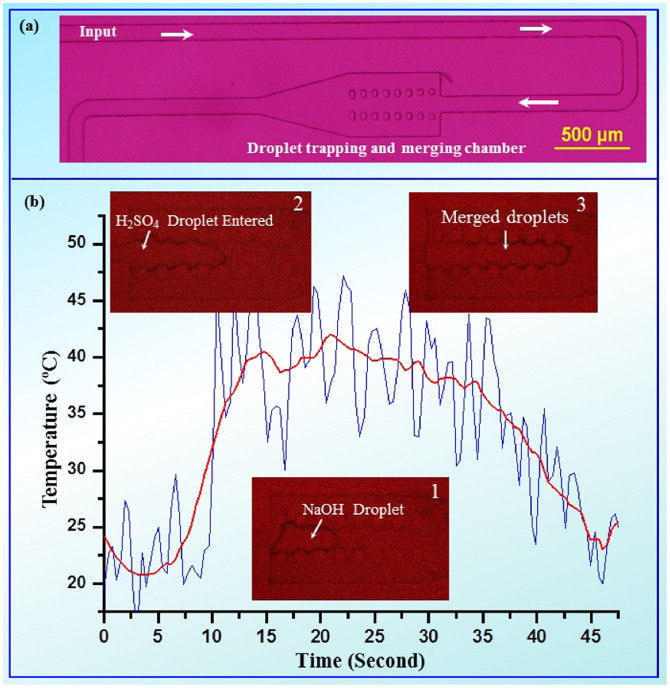
(a) Top view of the chip for droplet trapping and merging. (b) Variation of temperature during 3 nL NaOH droplet merging with 3 nL H_2_SO_4_ droplet. Red line is smoothed data by adjacent-average. Three insets (1 ~ 3) were taken via focusing on the droplet.

Additionally, we studied the local heat production and dissipation by an interior exothermic droplet reaction in micro-scale. A 3 nL NaOH and a H_2_SO_4_ droplets were generated by sampling device and then merged in an RAP chip (Figure 4a). The temperature variation during NaOH droplet merging with H_2_SO_4_ droplet are given in Figure 4b.

It is observed that considerable noise is presented in the measurement which is mainly caused by the usage of relatively large pixel size and high image capturing rate. Nevertheless, it can eventually provide down to 10 ms temporal resolution for dynamic investigation of droplet reaction if we reduce the observation area. The sudden rise of temperature to the maximum (about 42°C) in a few seconds is induced by two exothermic reactions: neutralization reaction between acid and alkali, and dilution of sulfuric acid. These two reactions may happen alternately during droplets fusion and mixing, which gave rise to the fact that the temperature stayed for a few seconds and then decrease slowly to RT. In comparison, we have done a bench-top experiment in mL volume. The total energy released from a nL droplet reaction reduces dramatically one million times comparing to bench-top experiment in mL volume. In the meantime, the SAV ratio of the droplet is about 30 times larger than that of bench-top size mL droplet, which determines the effective heat loss area and heat-releasing rate. The maximum temperature of the bench-top experiment is 74°C, which is almost doubled than that of the droplet reaction. Strikingly, we found that the heat-releasing time (from the reaction begin to temperature down to RT) of the micro droplet reaction (35 seconds) is much larger than that of bench-top size droplet (~12 min). We attributed the short heat-releasing time to the rapid mixing, small volume and large SAV ratio of the droplet reaction.

## Discussion

The aforementioned temporally-and-spatially temperature mapping results confirmed that our RAP functional material, combining with the confocal microscope, is suitable for mapping 3D thermal propagation in microfluidics. Comparing physically mixed RhB and PDMS, the chemically bonded RAP provides a more thermal-, photo- and solvent- stable and temperature sensitive functional material. This in turn assured the micrometer (5 μm) spatial and millisecond (10 ms) temporal resolution of our 3D temperature mapping approach based on the confocal microscope and the fluorescence quantum efficiency of RAP. The FEM simulated results support our experiments consistently especially for axial temperature distribution profile. A more accurate and precise 3D temperature mapping in microfluidic chip might be obtained by ratiometric fluorescence techniques which have been previously employed by others^24^,^25^,^26^.

In particular, this 3D thermal visualization technique was adopted to probe the local temperature and heat transfer of nL droplets. The heat inside a single droplet was produced in two ways: externally from IR absorption and internally from physical and chemical reactions. The external way is artificially controlled by the IR laser and the unsteady and steady heat transfer was studied by the real-time temperature detection. The heat energy of the droplet was pumped wirelessly by IR laser and could be calculated, therefore the heat transfer could be further qualified in such a micro-environment. Much huger heat energy could be harvested by the tiny droplet through raising the IR laser power and more aggressive and attractive behaviors such as explosion could be observed in this system. The latter way is passive. The heat released or transferred in droplet is complex typically involving the reactions energy which is governed by the volume and ratio of the reactants and the mixing, and interfaces which is determinate by the properties and structures of ambient oil and RAP. In the form of droplet, the reaction volume (droplet volume and number) could be digitalized by our micro-sampling technique; the droplet mixing is rapid due to the short diffusion length and therefore it can be quantified by dye fusion; the reaction volume and effective heat loss area of droplet is much smaller and larger than bench-top experiment, respectively. Even the aggressive and explosive reactions can be actualized and studied microscopically in droplet, which in turn promote the safety and maneuverability. Hence, this type of micro-reaction is much more controllable and the heat energy could be digitalized, transported and released in the form of droplet.

Finally, PDMS is the primary material for inexpensive microfluidic chip fabrication so far, owning to fast and easy micro-fabrication routines^27^,^28^. PDMS-based functional materials are of great scientific interest and importance for increasing the functionality and component of microfluidic chip and carrying out all the processes within a single chip from only a few square millimeters to centimeters in size^29^. Our RAP microfluidic chip exhibits mechanical reliability and our unique chemical grafting process is compatible with other PDMS-based functional materials (such as silver or carbon-PDMS for electrical input or sensing^30^,^31^, and carbonyl iron-PDMS for actuation^32^), all of which can be assembled into one chip by soft lithography for multifunctional chip. Such strategy is strongly desirable for the highly increased density and complexity (from 2D to 3D) of structure associated with miniaturization tendency in microfluidics chip.

In summary, we have established a method for imaging the 3D thermal distribution inside microfluidic chip by performing optical sectioning of our novel RAP. This new temperature mapping technique offers micrometer spatial and millisecond temporal resolution. Furthermore, it has been integrated with droplet generation and manipulation techniques seamlessly, providing a new, rapid and exquisite methodology to investigate the droplet reaction. From our initial experimental investigations and COMSOL simulation, surrounding local temperature could be used to probe the localized unsteady and steady heat transfer and thereby aid future discoveries of the heat generation, transfer and even transport mechanisms in the ingenious style of tiny droplet.

## Methods

To prepare the RAP functional material, there are three major steps. First, PDMS, AGE and RhB are mixed together. PDMS is produced with Sylgard 184 silicone elastomer mixture (Dow Corning Corporation, Miland, USA) at a weight ratio of Base:Curing agent = 10:1. The mixture is being left at room temperature for 3 hours, and then mixed with AGE and RhB solution homogeneously. To be noted that RhB could not be dispersed in PDMS directly, therefore RhB solution is prepared in ethyl alcohol and cyclohexane mixture (volume ratio = 3:1). With this method, RhB is being solubilized in PDMS by cyclohexane and is maintained at 0.02 wt% in PDMS. Ethyl alcohol, cyclohexane and the bubbles generated during blending were then removed by vacuum. Several repetitions of blending and vacuuming produced a uniform red gel-like RAP mixture which is well-prepared for curing, the second step. The curing time is approximately 36 hours at room temperature. The final step would be incubating the RAP functional material in an oven at 100°C for different duration.

For the chip part, 100-μm-high micro-channels and micro-pillar were fabricated by soft lithography^27^ inside the functional material. After the chip is bonded by plasma treatment, they were further incubated in oven at 100°C for 3 days in order to functionalize RhB to PDMS and reverse plasma-induced surface change which recovered microchannel surface to hydrophobicity.

Absorption spectra of RAP with 0.5 cm thickness were measured on Perkin-Elmer UV/VIS spectroscopy (Lambda 20). Fourier Transform Infrared (FT-IR) analysis was performed on Bio-Rad FT-IR Spectroscopy (FTS 6000) using attenuated total reflection technique (GladiATR, PIKE). Fluorescence spectra of RAP were carried out on Perkin-Elmer Luminescence Spectrometer (LS-55) excited at 514 nm.

RAP was coated on a double-sides-polished Si wafer (1.5 cm × 1.5 cm × 400 μm) by spinning (6000 rpm, 60 seconds) with 5 μm thickness. The Si wafer was attached to an aluminum home-made thermal stage. The temperature dependence of fluorescence was calibrated with a K type thermal couple. The fluorescent images were taken the confocal laser scanning microscope (Leica TCS SP5) through objectives with 10× and 20× magnification (numerical aperture = 0.30 and 0.4). Average intensity was measured from fluorescent images for temperature calibration. Argon Laser at 514 nm was maintained on 5.75 mW. It is also worth noted that the laser power stability has great influence to the accuracy and precision of temperature sensing, so all fluorescence intensity was measured after at least half hour of stabilization. Since thermal expansion (including heating stage, Si wafer and RAP) unavoidably led to focus drift, at least three markers in RAP were made for alignment. The relative intensity was plotted as a function of temperature and time in Figure S1 (b–c).

To heat the droplets inside the RAP microfluidic chip, a device setup was constructed (Figure S2). The droplets generated by sampling device were introduced into RAP chip. Detailed technical information of the sampling device could be found in our previous paper^4^. The IR laser was 3W with maximum emission at 980 nm which is not absorbable by RAP (Figure 1a). The IR laser was focused on RAP chip by a convex lens and they were aligned with the help of up-conversion nanoparticle which absorbed IR and emitted green light. RAP chip offered a place for droplet manipulation (trapped or merged). Two black ink droplets with 2.8 and 5.3 nL were trapped in RAP chip (Figure 2a). The IR laser was manually turned on to heat the black ink droplets. The fluorescent sections were taken at different places under droplets along z direction with the confocal microscope. The nearest section from droplet was defined as 0 and the distance increased along z direction. The average temperature of an area with 10 μm diameter in the center of the section was defined as central temperature. The central temperature under the droplets was measured according to the fluorescent variation. The fluorescent image was taken at 520 ms.

The NaOH (10 wt%) and H_2_SO_4_ (49 wt%) droplets with 3 nL were generated by sampling device and then merged in RAP chip. Figure 3a shows the full view of the RAP chip for droplet trapping and merging. The merging chamber is about 7 nL which is larger than the total volume of these two droplets. Therefore droplet was passively trapped in the chamber after coalescence^33^. The temperature variation was measured before and after merging two droplets. The fluorescent image was taken at 73 ms.

## Author Contributions

J.W., T.Y.K. and W.W. contributed to the experiments, interpretation, and writing. X.L. and J.W. contributed the simulation. W.C., Y.W., J.H., Y.H. and D.Z. provide experimental and writing guidance. All authors reviewed the manuscript.

## Supplementary Material

Supplementary Information Supplementary Info
