# Supplementary material for: Mapping three-dimensional temperature in microfluidic chip
Source: Sci Rep. 2013 Nov 25;3:3321. doi: 10.1038/srep03321 (PMC3839638; doi:10.1038/srep03321)
Supplement: Supplementary Information — Supplementary Info [file srep03321-s1.doc]

**Supplementary Information**

**Mapping three-dimensional temperature in microfluidic chip**

Jinbo WU^1^, Tsz Yan KWOK^1^, Xiaolin LI^1^, Wenbin CAO^2^, Yu WANG^1^, Junying HUANG^1^, Yaying HONG^2^, Dongen ZHANG^1^ and Weijia WEN^2^*

1 Department of Physics, the Hong Kong University of Science and Technology,

Clear Water Bay, Kowloon, Hong Kong

2 Nano Science and Nano Technology program and Department of Physics, the Hong Kong University of Science and Technology, Clear Water Bay, Kowloon, Hong Kong

Correspondence and requests for materials should be addressed to W.J. WEN (email: phwen@ust.hk)

**
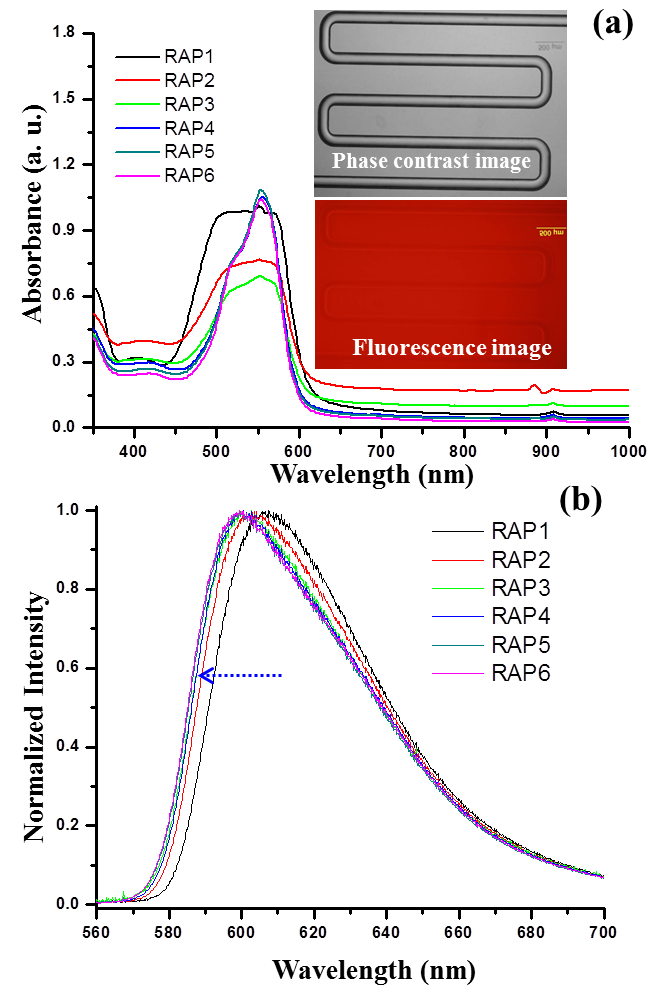
**

**Figure S1** Optical characterization of RAP (a) UV-Vis spectra of RAP 1~6. Inset is phase contrast and fluorescence images of RAP microfluidic chip. (b) Fluorescence emission of spectra of RAP 1~6. RAP 1~6 with 0.5 wt% of AGE are represented by 0, 5, 10, 15, 20, 25 hours of incubation time at 100 °C.


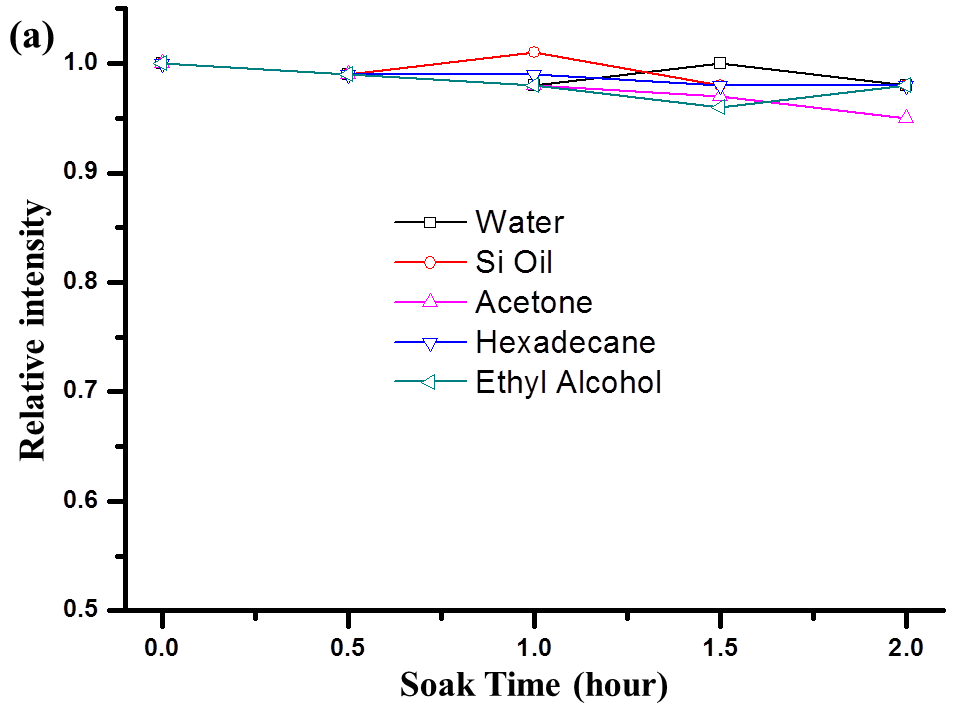

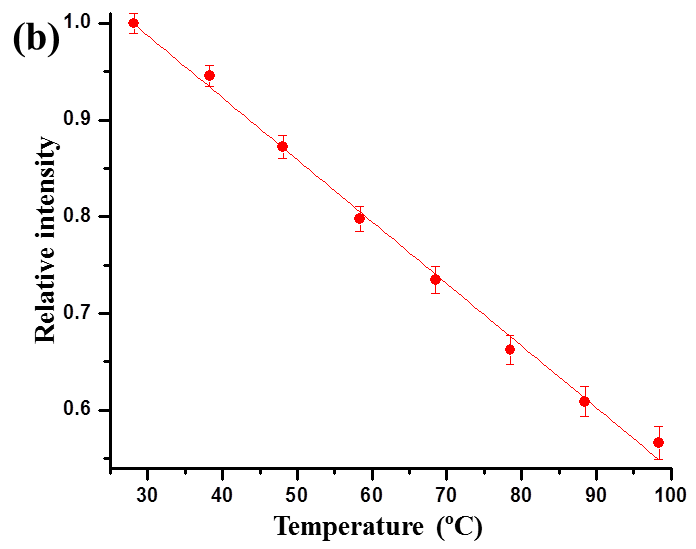

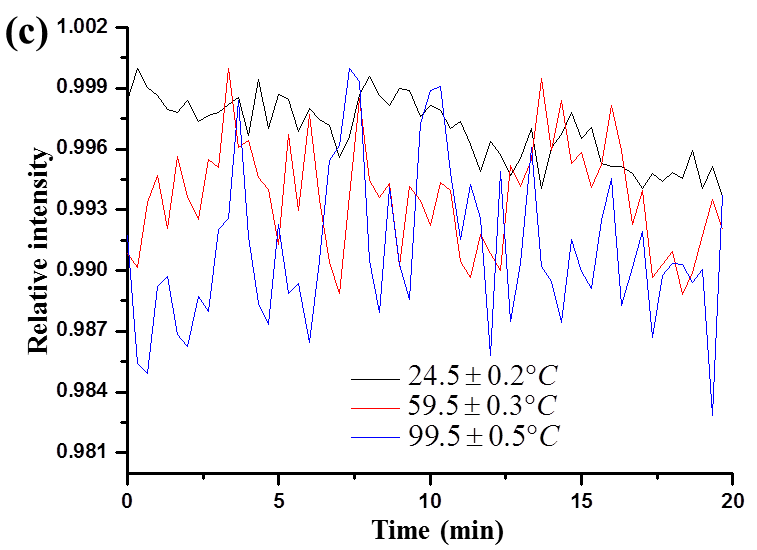


**Figure S2**. (a) Solvent soaking experimental result. Relative fluorescent intensity was plotted as function of soak time of RAP. 0.1 g RAP was immersed into 1.5 ml of five common solvents and then the fluorescent intensity of RAP was measured at different times under the same excitation condition. (b) Normalized fluorescence intensity as a function of temperature for calibration of the fluorescence-based temperature measurement. (c) Normalized fluorescence intensity as a function of time at three temperatures for photostability and thermal stability testing. The intensity in Supporting Figure S2 b-c is average intensity of a whole fluorescent picture in the size of 512×512. So those are not the intensity of one pixel but the average intensity of 262514 pixels. Figure S2 b-c show that the average intensity is stable. In other word, the intensity in most of the 262514 pixels is stable but some of the pixels may fluctuate and those are the noise. Currently, the pixel size is 1.52 µm in our confocal microscope (Leica TCS SP5). It always needs at least 3 pixels to distinct clearly one object that is
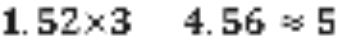
 . So the spatial resolution was estimated to be 5 µm.


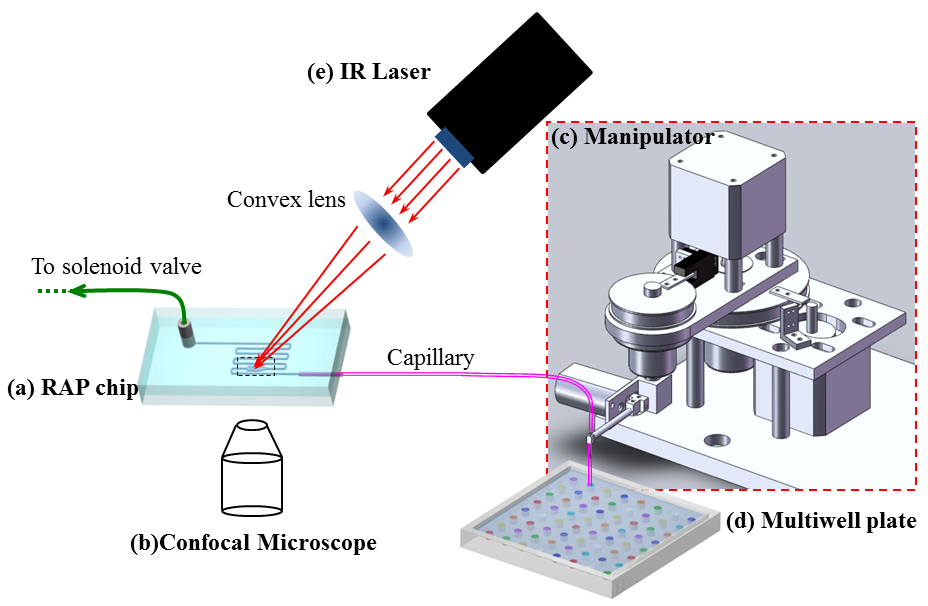


**Figure S3.** Droplet thermal manipulation device setup: (a) RAP chip, (b) confocal microscope, (c) manipulator, (d) multiwall plate and (e) IR laser.


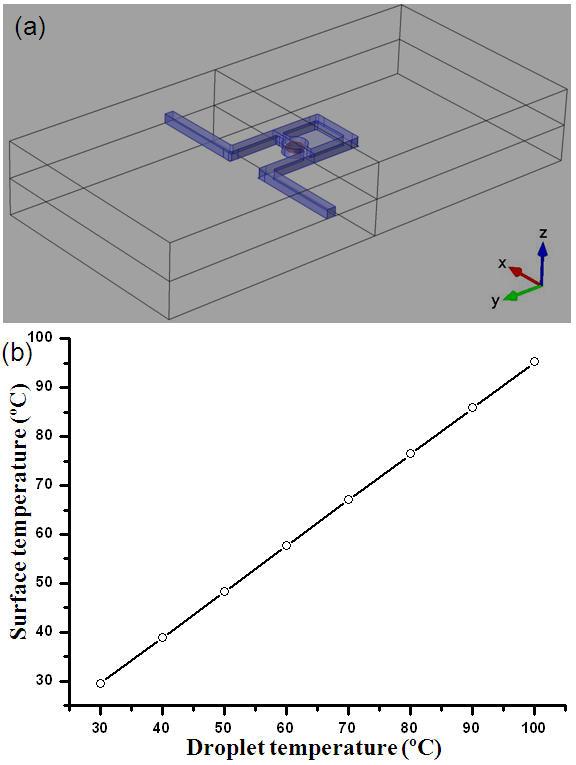


**Figure S4.** (a) The 3D geometry (only 2.8 nL droplet) of microfluidic chip was simplified and cut out. We assumed that there is 5-µm gap between the droplet and up/down channel surfaces, and the temperature of the droplet and chip surface (room temperature) is homogeneous and constant. Under this assumption and the physical parameters in Table S1, Heat Transfer (solid and liquid) module in COMSOL Multiphysics is used to solve the time dependent equations. (b) Temperature difference between the droplet and surface (the highest central temperature).


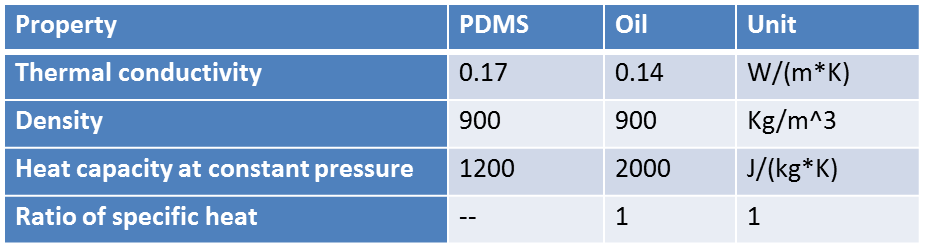


**Table S1.** The physical parameters of PDMS and Si Oil for FEM simulation in COMSOL.
